# From Medical Imaging to Bioprinted Tissues: The Importance of Workflow Optimisation for Improved Cell Function

**DOI:** 10.1017/erm.2025.10018

**Published:** 2025-09-12

**Authors:** Jesús Manuel Rodríguez Rego, Laura Mendoza Cerezo, Francisco de Asís Iñesta Vaquera, David Picado Tejero, Alfonso Carlos Marcos Romero

**Affiliations:** 1 Departamento de Expresión Gráfica, Escuela de Ingenierías Industriales, Universidad de Extremadura, Badajoz, España; 2 Departamento de Bioquímica y Biología Molecular y Genética, Facultad de Ciencias, Universidad de Extremadura, Badajoz, España

**Keywords:** 3D bioprinting, cell viability and function, functional hydrogels, image processing, modelling, personalised medicine

## Abstract

**Background:**

The rapid advancement of 3D bioprinting is transforming possibilities in tissue engineering and personalised medicine, offering innovative solutions to critical biomedical challenges such as organ shortages and the need for precise 3D cellular models. To fully unlock the potential of this technology, anoptimised and comprehensive workflow is essential.

**Methods:**

This review provides a systematic examination of the bioprinting process, covering key steps from medical image acquisition to the validation of bioprinted structures. The analysis includes biomaterial and cell type selection, conversion of DICOM images into 3D-printable models, and slicing techniques.

**Results:**

Key factors influencing the precision, viability, and clinical relevance of bioprinted tissues are identified. Comparisons between planar and non-planar slicing algorithms highlight their impact on scaffold integrity. The review also discusses advancements in algorithm development, bioprinter technology, and biomaterial optimisation, emphasising their role in enhancing reproducibility and functionality.

**Conclusions:**

This structured review offers actionable insights for researchers and practitioners aiming to refine bioprinting workflows. By integrating improvements across imaging, modelling, and material selection, 3D bioprinting can more effectively support the development of clinically relevant constructs, advancing regenerative medicine and personalisedhealthcare.

## Graphical abstract

**Figure:**
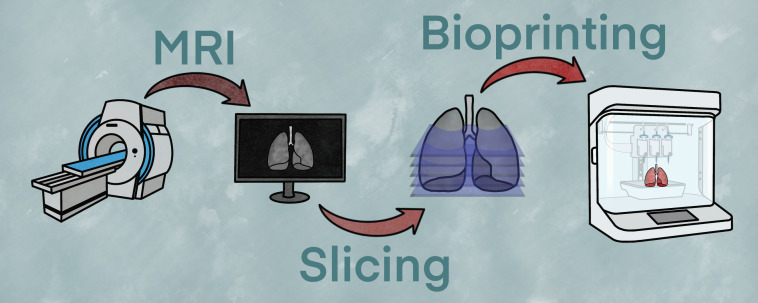

## Introduction

One of the most significant challenges currently facing modern medicine is the lack of effective and scalable technologies for tissue regeneration. Major challenges include organ shortages: despite tireless efforts and diverse medical and political initiatives, the number of organs available does not meet the growing demand of patients waiting for transplantation (Ref. 1). The World Health Organisation estimates that only 10% of the global demand for transplantation is met (Ref. 2), while the *United Network for Organ Sharing* reports that, in the United States, an average of 20 people die every day waiting for an organ transplant (Ref. 3). In addition, research on novel therapies is hampered by a lack of suitable study models to test their efficacy (Ref. 4). For example, 2D monolayer cell cultures do not recreate tissue molecular mechanisms occurring *in vivo*, due in part to a lack of realistic physiological conditions (Ref. 5). As a result, there is limited correlation of 2D culture results with real-life in vivo scenarios (Ref. 6).

To overcome these limitations, scientists are developing 3D cultures that can offer advanced test and /or organ systems with realistic tissue microenvironments. These 3D cultures have been developed in parallel with bioprinting, an additive manufacturing technology that prints living cells in a pre-designed pattern (Ref. 7). Additive manufacturing generates a three-dimensional structure by adding cross-sections in superimposed layers, without the need for a pre-existing mould. The advanced 3D cell cultures generated with this technology may potentially leads to the generation of viable tissues and organs for transplantation to patients in need, producing major advances in the field of personalised medicine.

Unlike conventional cell seeding approaches, encapsulation during deposition enables a homogeneous and spatially controlled cell distribution, which is difficult to achieve with standard seeding methods that typically result in peripheral colonisation and oxygen gradients towards the core of the construct (Ref. 8). Moreover, direct bioprinting allows for the simultaneous deposition of multiple cell types and vascular channels, making it possible to generate perfusable tissues several millimetres thick that can be maintained for weeks (Ref. 9), while the bioink provides a biomimetic three-dimensional microenvironment that supports cell viability and function (Ref. 10). These advantages justify the use of direct cell bioprinting, while acknowledging that cell seeding onto pre-printed scaffolds remains valuable when materials or conditions are not compatible with the printing of living cells.

There are already numerous examples demonstrating the potential of bioprinting in both clinical and preclinical settings, with successful outcomes in animal models and in humans. In animals, studies have reported the implantation of facial constructs in rats (Ref. 11), functional skin grafts containing six different cell types (Ref. 12), meniscus substitutes in rabbits (Ref. 13), bioprinted muscle tissues implanted in rats (Ref. 14), as well as bone implants (Ref. 15), cardiac patches (Ref. 16) and oesophageal substitutes (Ref. 17), all of which showed tissue integration and partial or complete functionality. In humans, a notable example is the use of autologous dermal fibroblasts bioprinted for nerve injury repair, opening the way to direct clinical applications (Ref. 18). These advances highlight that bioprinting is beginning to bridge the translational gap, although regulatory and standardisation challenges remain before widespread clinical adoption can be achieved.

Despite remarkable progress, bioprinting still faces significant challenges for clinical translation. Among the technical hurdles are the scalability of constructs and the need to generate clinically relevant tissues that maintain cell viability, the incorporation of efficient vascularisation to overcome diffusion limits and enable stable perfusion in thicker tissues (Ref. 19), and the mechanical stability of scaffolds and bioinks to withstand long-term physiological loads (Ref. 20). In addition, regarding clinical application, barriers remain due to the absence of specific regulations for cell-laden bioprinted products, which must currently be classified under the framework of advanced therapy medicinal products in the EU (Ref. 21) and the FDA’s guidelines for additive manufacturing (Ref. 22). Likewise, the lack of standardisation in bioink characterisation and interlaboratory variability continue to limit the reproducibility of results (Ref. 23).

This review examines the complete workflow involved in some of these emerging technologies. Every approach should meet the requirements for their intended biological function, such as appropriate bioprinting post-processing and improved cell viability within the generated structures. We discuss advanced image processing and slicing techniques, which are essential for accurately translating patient-specific anatomical data into three-dimensional bioprinted models. Finally, this review highlights the role of medical imaging in optimising the precision and viability of bioprinted scaffolds, thereby advancing the potential of 3D bioprinting for personalised medicine and organ transplantation.

## Types of bioprinting

3D bioprinting encompasses a diverse set of technologies that enable the spatially precise deposition of living cells and bioinks to generate biomimetic structures. Each modality is based on distinct physical principles and offers different advantages and limitations. The main bioprinting approaches are described below, together with their most relevant benefits and drawbacks, as well as representative examples of in vivo success that illustrate their translational potential.

- **Extrusion-based bioprinting (EBB):** This technique employs pneumatic or mechanical pressure to dispense biological inks. It is an affordable and widely used method capable of fabricating cells, tissues, organ-on-a-chip systems and even human-scale constructs, with several commercial platforms currently available (Ref. 24). Its main advantages include the ability to print highly viscous bioinks with elevated cell density, to deposit continuous filaments and large structures and to easily integrate multiple materials and gradients (Ref. 25). However, the shear stress generated in the nozzle during printing can compromise cell viability, and the resolution is generally lower than that of light- or laser-assisted systems (Ref. 26). At present, extrusion-based printing remains one of the most widely applied technologies in biomedical research and development (Ref. 26). A representative example is the in situ bioprinting of autologous fibroblasts and keratinocytes into a stratified skin-like structure in murine and porcine wound models, which resulted in improved wound healing (Ref. 27).
  Beyond conventional extrusion strategies, emerging methods such as FRESH and SWIFT have been developed to overcome current limitations and expand the potential of bioprinting:
  - **FRESH (Freeform Reversible Embedding of Suspended Hydrogels):** This approach employs a thermoreversible support bath that acts as a temporary matrix during printing, enabling free nozzle movement and controlled bioink deposition without structural collapse. It allows the fabrication of complex 3D constructs using low-viscosity bioinks while ensuring high structural fidelity and cell viability close to 99.7% (Ref. 28). FRESH is particularly advantageous for printing delicate materials such as collagen while preserving the architecture (Ref. 29), although it requires additional steps for bath preparation and removal (Ref. 30).
  - **SWIFT (Sacrificial Writing Into Functional Tissues):** This strategy generates vascular channels within functional tissue constructs by printing sacrificial gelatine inks at 4 °C into organ-building blocks composed of stem cell–derived multicellular spheroids embedded in a viscous matrix. Upon heating to 37 °C, the gelatine liquefies, leaving perfusable vascular networks that confer functionality to the tissue (Ref. 31). Compared to other approaches, SWIFT enables the fabrication of organ-specific tissues with high cell density and integrated vascular channels, yielding viable and functional constructs (Ref. 32). However, to maintain high fidelity, the sacrificial filament diameter must be approximately 400 μm, about twice the size of the spheroids used (Ref. 33).
- **Inkjet-based bioprinting:** In this modality, the bioink – comprising a hydrogel prepolymer mixed with encapsulated cells – is loaded into a cartridge connected to a printhead. Thermal or piezoelectric actuators electronically deform the printhead to eject controlled droplets of bioink during printing (Ref. 34). Inkjet printing offers high speed, precision, resolution, cost-effectiveness and the ability to deposit different cell types or biomaterials within the same construct. Nonetheless, concerns remain regarding thermal and mechanical stresses, which may affect gene expression and cellular biochemical processes in sensitive cells (Ref. 35). Using this technology, bioprinted skin implants have been successfully produced in murine models (Ref. 36).
- **Light-assisted bioprinting (stereolithography):** This approach relies on light-induced polymerisation of a photosensitive bioink, enabling rapid fabrication of tissue structures by solidifying entire layers at once (Ref. 37). A major limitation is the use of UV light to activate photoinitiators commonly added to bioinks, which can negatively impact encapsulated cells. Recent progress, however, includes the development of visible-light photoinitiators to mitigate these effects (Ref. 38). Light-assisted bioprinting has been successfully applied to produce scaffolds for spinal cord injury repair in murine models (Ref. 39).
- **Laser-assisted bioprinting (LAB):** This technique employs pulsed laser energy directed at a multilayer ribbon containing bioink. The laser induces vaporisation of a thin metallic layer, generating bubbles that propel bioink droplets towards a substrate with culture medium. LAB enables high-resolution deposition of cells and hydrogels, with resolutions ranging from picometers to micrometres depending on bioink properties, laser parameters and substrate conditions (Ref. 40). Advantages include high precision, reduced risk of nozzle clogging, deposition of high-density cell suspensions and droplet generation rates up to 5000 per second, with demonstrated potential for in situ printing in animal models and combination with other technologies (Ref. 41). Nonetheless, LAB faces limitations in productivity, usability and sustained operation, as technical issues often arise after only a few minutes of printing. Furthermore, cellular homogeneity can increase gravitational settling, sometimes requiring additives such as glycerol for suspension stability (Ref. 42). This technique has been successfully applied in bone tissue regeneration in murine models (Ref. 43).
- **Microfluidic bioprinting:** This technology integrates engineering, physics, chemistry and biotechnology principles to manipulate minute volumes of fluids, cells and biomolecules within microchannels. It enables the fabrication of complex, heterogeneous 3D tissues that more accurately replicate native tissue organisation and functionality (Ref. 44). Microfluidics provides precise control of flow, mixing and bioink positioning, reduces shear stress through laminar sheath flow and enhances morphology and resolution, particularly when combined with extrusion-based printing, extending resolution beyond the ~50 μm limit of microextrusion (Ref. 45). Challenges remain in scaling up tissue structures, handling fragile hydrogel fibres and achieving consistent geometric outcomes (Ref. 46). A notable example is the development of a glioblastoma-on-a-chip model that realistically recapitulates tumour ecology, including cellular heterogeneity, ECM complexity, oxygen gradients and dysfunctional vascular interactions, thereby enabling patient-specific therapeutic resistance profiling and drug prioritisation (Ref. 47).
- **Hybrid and multimaterial approaches:** These strategies involve the sequential or simultaneous deposition of two or more bioinks, which may be single-component, homogeneous multicomponent or multiphasic composites, to impart specific properties and functionalities to different regions of the construct (Ref. 48). Natural biomaterials such as collagen, fibrin, chitosan, alginate, gelatine and hyaluronic acid provide high biocompatibility but suffer from limited mechanical strength and stability, motivating their combination with synthetic materials such as PCL, PEEK, PLA, PEG or Pluronic, which enhance consistency and mechanical performance in multilayered constructs (Ref. 48). Two main strategies are typically pursued: (i) pre-mixing materials to improve printability and mechanical properties, or (ii) integrating distinct structures to combine the individual advantages of each material in terms of strength, resolution, processability and cost (Ref. 49). Multimaterial scaffolds can be fabricated using separate cartridges, multiscale structuring, or surface modifications with specific chemistries, resulting in improved in vitro and in vivo performance (Ref. 50). Limitations include dependency on restricted combinations (extrusion with electrospinning or post-processing), limited vascularisation capacity, difficulty in finely tuning material properties during printing and challenges in scaling down to submicron or nanoscale dimensions (Ref. 50). Using a multimaterial approach with a microfluidic printhead coupled to a coaxial needle, muscle tissue was successfully mimicked and implanted in murine models, achieving partial muscle organisation in vivo with tissue integration and cell viability, although challenges remain in preserving muscle architecture after implantation (Ref. 51).

## Medical image acquisition and processing

The acquisition of medical images is the first step in the generation of personalised scaffolds by 3D bioprinting. This step allows detailed analysis of tissue anatomy and pathology and provides a solid basis for the design of biomimetic three-dimensional structures.

Medical imaging techniques include X-ray, CT, PET, magnetic resonance imaging (MRI), single photon emission computed tomography (SPECT), digital mammography and diagnostic ultrasound (Ref. 52). More recently, ultrasound has been successfully used in the cardiovascular field, including 3D transthoracic echocardiography (TTE) and transesophageal echocardiography (TEE). 3D rotational angiography has also been employed. In addition, the combination of different imaging modalities, such as CT and TEE, allows the creation of hybrid 3D models that capture both structural and valvular morphology (Ref. 53).

The biological nature of the tissue of interest will dictate the selection of a particular imaging technique. For example, in the case of computed tomography (CT), tissue densities are directly related to pixel intensities, allowing the analysis of structures with high (e.g. bones) and low (e.g. lungs) densities, while MRI is more appropriate to appreciate differences between soft tissues (such as the white and grey matter of the brain) (Ref. 54).

CT is an imaging technique that uses iterative algorithms to reconstruct the interior of large volumes using graphics processing units (GPUs) (Ref. 55). Due to its higher resolution and its ability to provide information about different tissue densities, it is a more interesting technology for medical imaging for bioprinting, as it facilitates both the generation of the 3D model and the choice of the appropriate biomaterial.

### DICOM image processing

3D bioprinting based on Digital Imaging and Communications in Medicine (DICOM) images starts with the conversion of 2D medical images, which are stacked, and are later processed to create a 3D printing compatible format (.STL). This process involves the segmentation of the DICOM images into a three-dimensional computer-aided design (CAD) format. From the data obtained, initial processing is performed, such as defining and adjusting the region of interest (ROI) (Ref. 56).

CT or MRI images are stored in DICOM format in the hospital’s Picture Archiving and Communication System (PACS) (Ref. 57) from where they are obtained for processing and subsequent bioprinting of the desired tissue. In case you want to use open sources, some such as *DICOM Library*, *TCIA* or *NIH Chest X-ray Dataset* contain numerous freely accessible DICOM files, which can be downloaded following the guidelines described by each.

The first step in converting DICOM images to .STL format is to improve the quality of the images through a thresholding and segmentation process using software that allows the CT image to be converted into a 3D model (Ref. 58).

A further improvement in the processing of DICOM images came from the use of virtual topologies combined with mesh optimisation algorithms. Recent studies have shown that these techniques make it possible to reduce the number of elements in the mesh, improving the resolution of the geometry without compromising the structural quality of the 3D model, which significantly reduces processing time (Ref. 59). This approach is particularly useful in complex geometries, such as airways, where anatomical details are critical for accurate simulation.

Some suitable software for this procedure includes *3D Slicer*, *OsiriX*, ITK-Snap, *Invesalius*, *MeshLab* or *Blender.* In the following, the use of *3D Slicer* will be explained because of its open-source status and its widespread use in DICOM image analysis.

#### 3D Slicer

*3D Slicer* is an open-source 3D data analysis and visualisation platform designed to facilitate the use of medical and scientific images. The software stands out for its ability to perform advanced segmentation and analysis, allowing users to segment medical images in two and three dimensions, which facilitates the identification and isolation of specific structures, as well as allowing the manipulation of volumetric and surface models in different planes, such as axial, sagittal and coronal.

It supports multiple data formats, such as DICOM, *Neuroimaging Informatics Technology Initiative* (NIfTI) or sections in .dcm format, allowing the integration of images from different modalities such as CT, MRI and ultrasound. In addition, 3D models can be exported in printable formats such as STL, facilitating the creation of physical models from medical images.

#### Using 3D Slicer to process medical images

**Figure 1.**
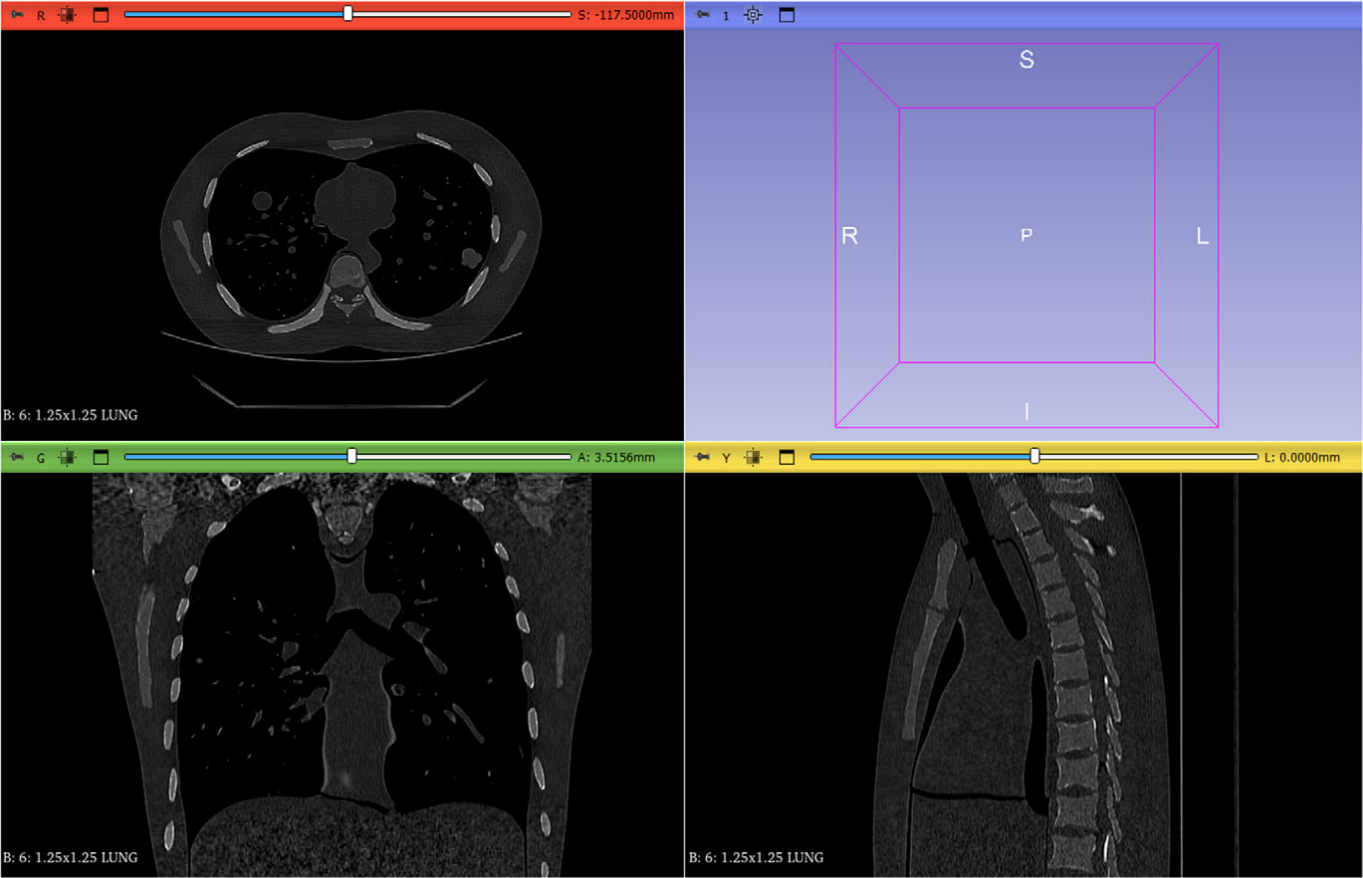
Detailed visualisation of the different anatomical sections.

Image processing begins by selecting the *Data* button and *Choose file(s) to Add* in the main 3D Slicer interface, from where it is possible to import individual DICOM files or entire folders containing multiple studies. In this case, to load a lung CT in .dcm format, all files corresponding to the sections must be selected. Once the files are uploaded, patient data such as patient name, ID, gender or year of birth, among others, can be viewed. The loaded file is then selected, and the *Load* option is chosen and the images automatically appear in the corresponding views (axial, coronal and sagittal), allowing a detailed visualisation of the different anatomical sections. In the case of Figure 1, a lung CT file has been chosen.

**Figure 2.**
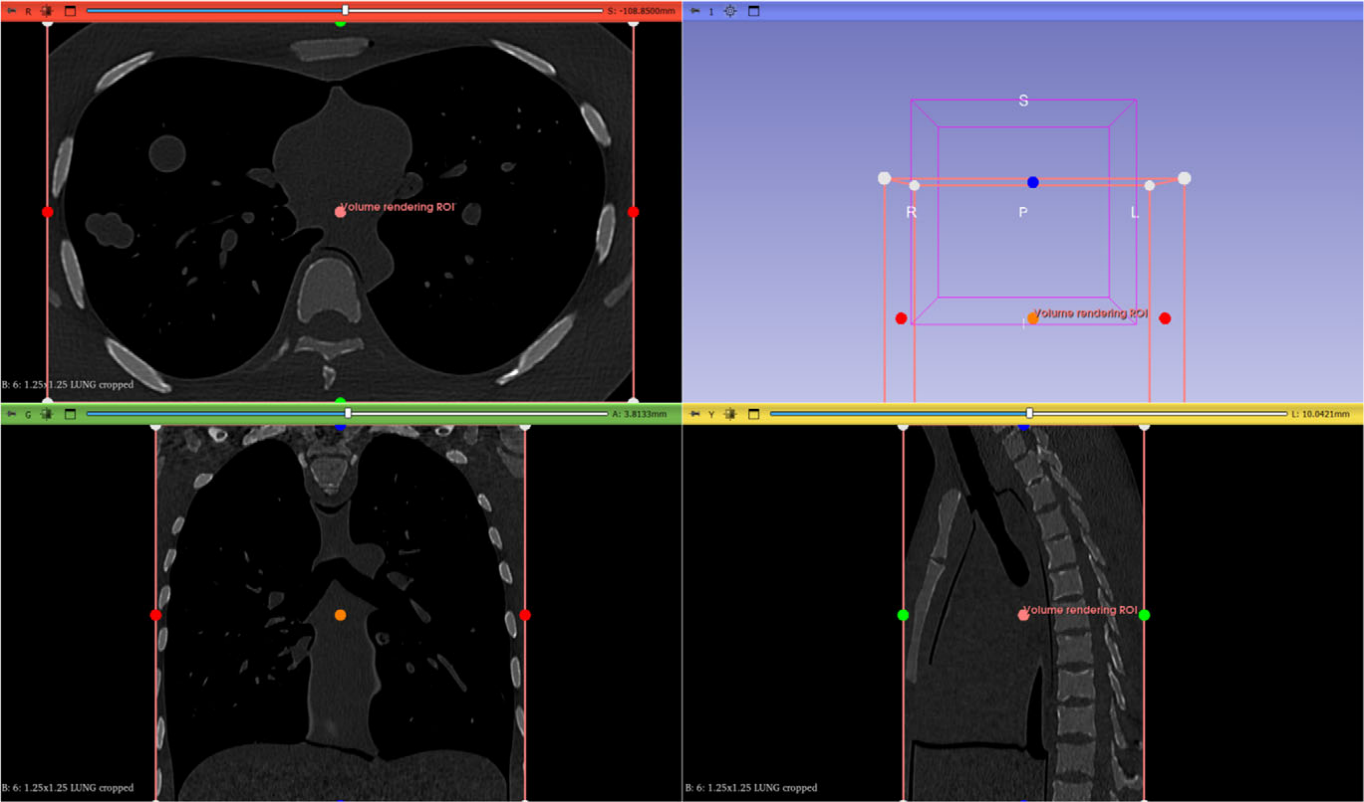
Selection of the desired area using the ROI (region of Interest).

Next (Figure 2), the desired area must be cropped using the ROI, choosing the *Volume Rendering* module, with the *Crop* option activated. By clicking on the Display ROI option (the eye) it is possible to delimit the area to be processed.

Subsequently, by searching the *Crop Volume* module, the delimited area can be accessed to facilitate segmentation, and after determining the cutting options, the *Apply* option is chosen. With this step, the area of interest is obtained for processing.

The next step involves adjusting contrasts to define areas that are not visible using the default settings via the *Volumes* module. The programme allows to analyse the images using default options, so in this case the *CT-Lung* option will be selected.

Next, the segmentation of the anatomical structures of interest must be carried out. To do this, access the *Segment Editor* module, where a new segment is created by choosing the *Add* option, on which the available segmentation tools will be applied (Table 1):

**Table 1.** Description of the tools available in 3D Slicer

Tool	Description
Threshold	Selects image regions based on a specific range of pixel intensities. Useful for segmenting structures with different densities, such as bone or soft tissue
Paint	Allows the user to manually draw on the image to add or modify parts of the segmentation
Erase	Used to remove regions from an existing segmentation
Scissors	Cuts parts of the segment using predefined shapes (circle, rectangle, polygon, etc.) or following a drawn trajectory
Grow from seeds	Expands an initial segmentation (seeds) until the edges of other regions in the image are met
Watershed	Segments different regions based on the intensity differences between connected areas, using a propagation approach
Level tracing	Automatically defines a contour based on the intensity of the image in a slice plane
Fill between slices	Fills gaps between manually drawn segments in different image slices
Smoothing	Smoothes jagged segmentation edges to generate a more continuous and aesthetically pleasing contour
Logical operators	Allows Boolean operations between segments, such as combining, intersecting or subtracting segmented volumes
Islands	Detects and separates ‘islands’ of connected voxels in the segmentation, allowing you to remove unwanted small areas or keep only the largest structure
Margin	Adds or reduces a margin around an existing segment, expanding or contracting it as needed
Hollow	Converts a solid segment into a hollow structure, useful for preparing models for 3D printing that require thin walls
Mask volume	Creates a volume based on a segment, applying a mask that modifies the original volume according to the defined segment
Draw	Draw freehand or pre-determined shaped segments on images to add fine detail in segmentation

One of the most widely used tools is *Threshold*, which allows selecting and isolating anatomical structures based on the density or intensity values of the images, such as bone or soft tissues. To do this, in the generated segment, the density of the structure of interest (in this case, the lung) must be selected with this tool, dragging until the desired area is selected, and allowing it to be differentiated from the rest of the image. The selection will be validated with the *Apply* option. Threshold values can be interactively adjusted to ensure accurate selection of relevant structures. If necessary, manual adjustments can be made using tools such as *Paint*, *Erase* or *Scissors* to ensure optimal segmentation.

**Figure 3.**
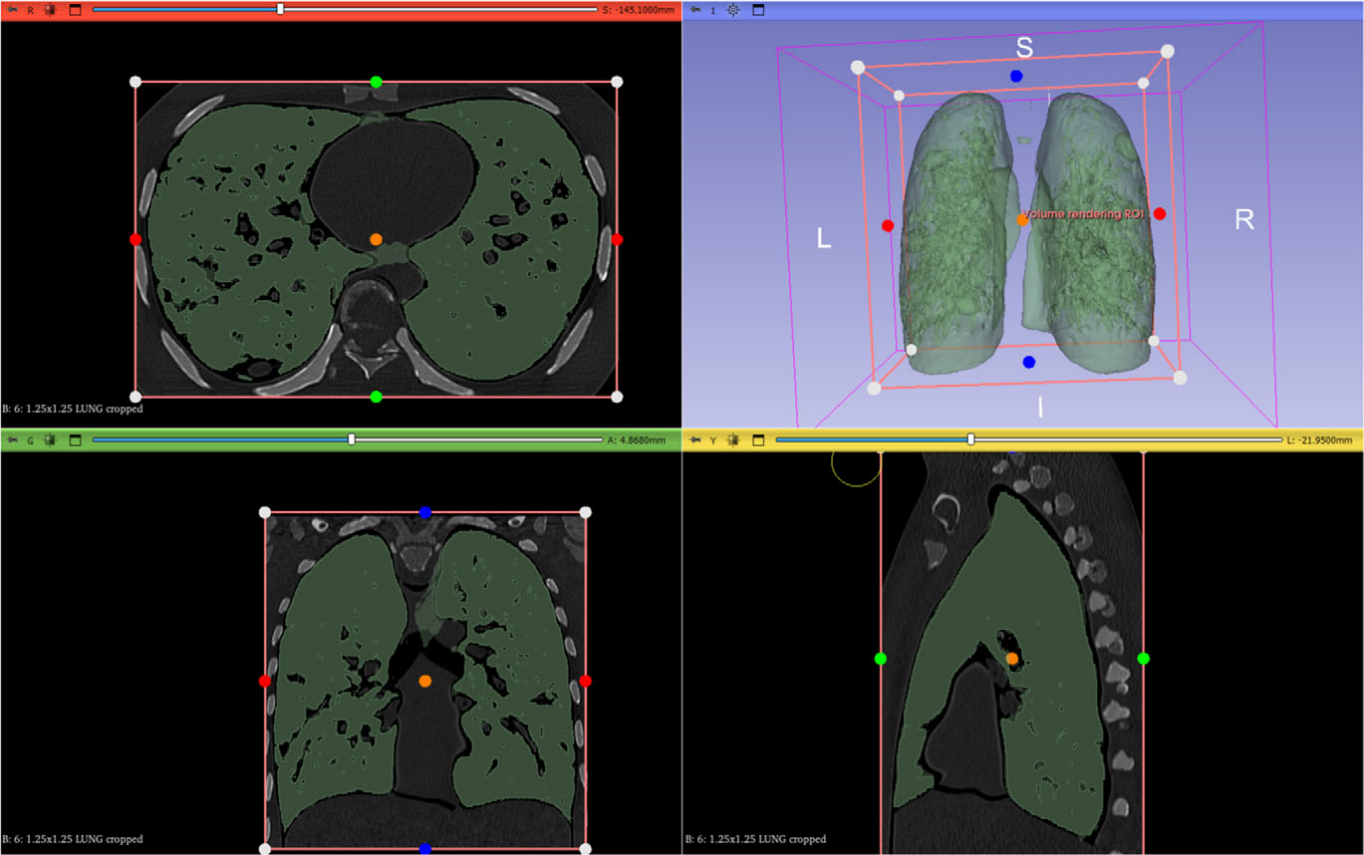
Isolation of the desired anatomical structure using the Threshold tool.

Using the *Show 3D* option, the result of the insulation of the structure can be observed after the necessary adjustments have been made (Figure 3).

Once the segmentation is finished, it is possible to improve the quality of the 3D model by applying smoothing algorithms through the *Smoothing* tool of the segment editor. This procedure reduces the irregularities present at the edges of the structures, improving the representation of the segmented surfaces. The level of smoothing can be adjusted according to the specific requirements of each case, providing precise control over the quality of the final model.

**Figure 4.**
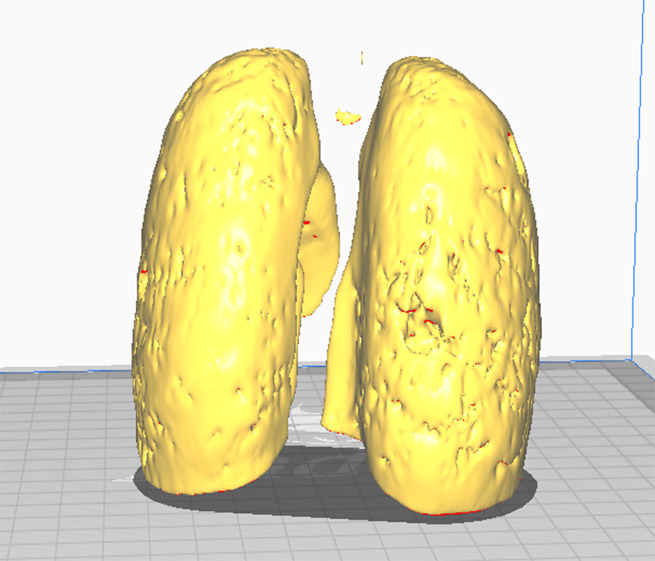
Export of the isolated 3D object obtained from the DICOM file to 3D printable .STL format.

When the segmentation has been refined, the next step is to convert it into a 3D model (Figure 4). This is done using the *Export to files* option within the *Segmentations* module. The STL format is one of the most common formats for this purpose.

Although 3D Slicer provides robust tools for STL model segmentation and generation, in some cases it can be useful to perform additional file optimisation prior to printing. This can be done using post-processing tools such as *Meshmixer* or *Blender*, which can repair errors in the mesh, simplify complex geometries and improve the structure of the model to ensure a successful print.

## Slicing and its impact on the final structure

For 3D models to be bioprinted, .STL files must go through two processes: slicing and optimisation of the bioprinting parameters. In 3D printing, slicing refers to the process of converting a 3D model into a series of 2D layers or slices that will be sequentially printed, using algorithms specifically designed for this function (Refs 60, 61).

### Types of slicing

The different slicing methods can be initially grouped into two families: planar slicing algorithm (flat) and non-planar slicing algorithm (out of plane or curved). Each of the different slicing techniques has advantages and disadvantages depending on geometry, type, amount of material and purpose.

#### Planar slicing algorithms

Printing occurs layer by layer, in one plane. They are the most common method of slicing because of their simplicity and because they are accessible to more types of technologies. However, they offer poor surface quality and require the use of supports so that the bioprinted structure does not collapse during printing, which increases material usage and decreases the speed with which we can obtain the final product (Ref. 62).

**Figure 5.**
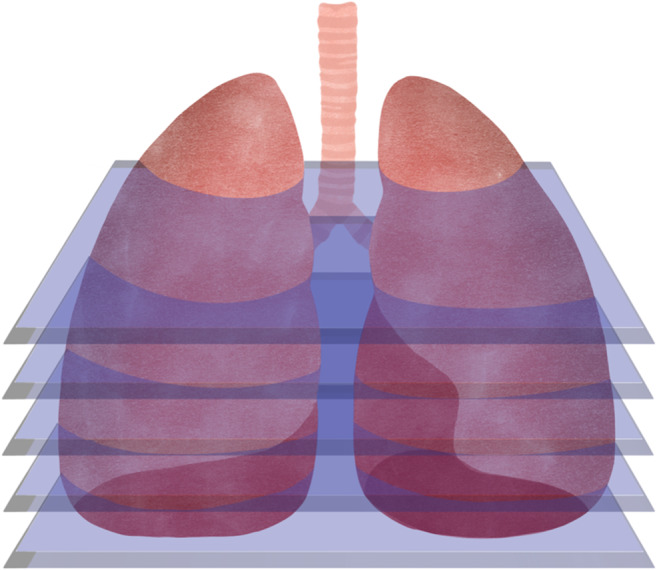
Uniform slicing by flat layers of the same thickness.

**Figure 6.**
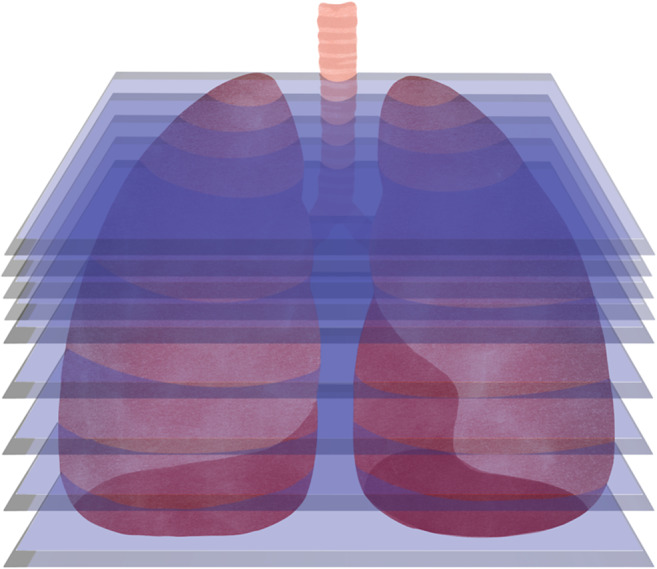
Adaptive planar slicing with layers of different heights.

This type of slicing considerably limits the performance of additive manufacturing (AM) systems, causing staggered surfaces, massive support structures, non-conformity with curved substrates and reduced strength in thin structures (Ref. 63). The most common types of Planar Slicing are:

- **Uniform slicing or Constant Layer Thickness Planar Slicing (Figure 5):** the most common method in 3D printing, in which the 3D model is cut into thin flat layers of the same thickness, which are deposited one by one by the printer (Ref. 63). The height of the layers is a key factor in the quality of the printed object, as the thinner the layers, the higher the resolution and detail of the final object. However, this flat layer-based cutting considerably limits the performance of AM systems, leading to staggered surfaces, massive support structures, non-compliance with curved substrates and reduced strength in thin shell structures (Ref. 64).
- **Adaptive Planar Slicing (Figure 6):** individual layers of the same part can have different layer heights, so details can be produced with high resolution, while non-detailed areas are produced with higher layer heights, improving production speed (Ref. 65) although it only improves the representation of curved surfaces. In addition, because the part is still made of flat layers, the staircase effect is not eliminated. It represents an optimisation compared to the previous method, as it allows a better surface finish to be obtained by reducing the staircase effect, with an optimised printing time (Ref. 65), and requires a much longer planning and adjustment stage than the previous techniques described (Ref. 66).

Almost all fused deposition modelling (FDM) bioprinters on the market are capable of printing from planar sliced 3D models, as these are the simplest slicings. The minimum requirements are that the extrusion can be performed in an XY plane and that they have 3 Degrees of Freedom (DOF). Examples of models that allow printing from planar sliced 3D models are NovoGenTM MMX Bioprinter, developed by Organovo and BIO X by CELLINK.

#### Non-planar slicing algorithms

**Figure 7.**
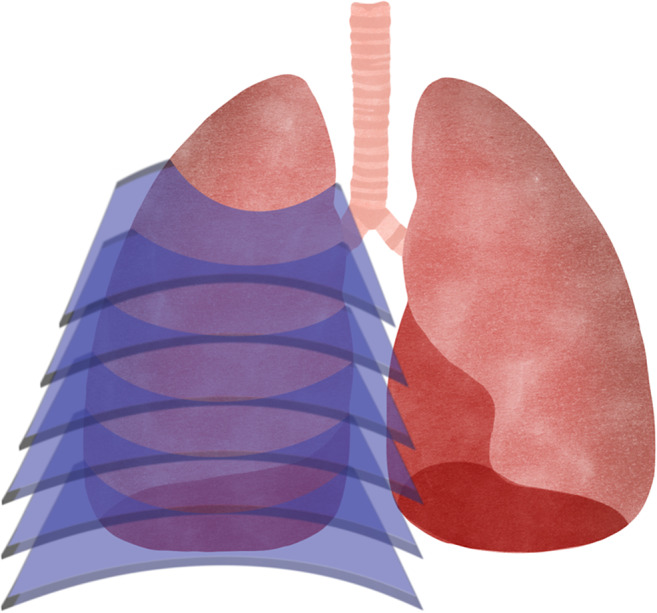
Curved-layer slicing.

**Figure 8.**
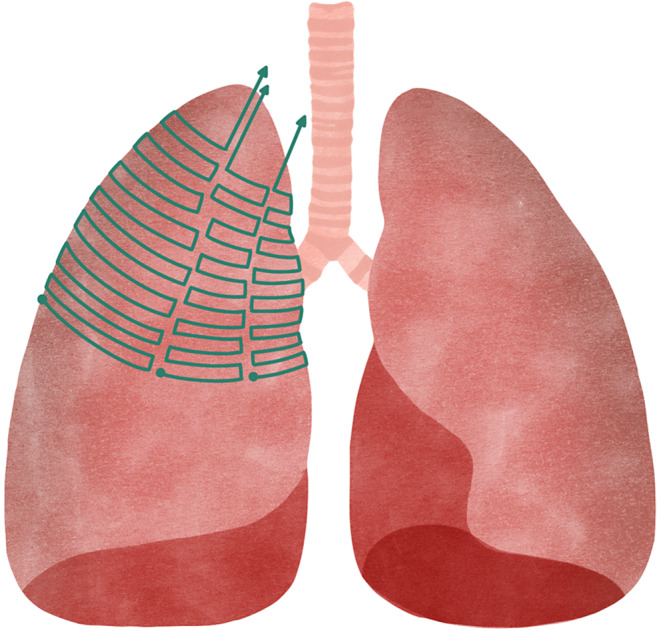
Conformal slicing where the cuts are carried out on a previously existing surface.

**Figure 9.**
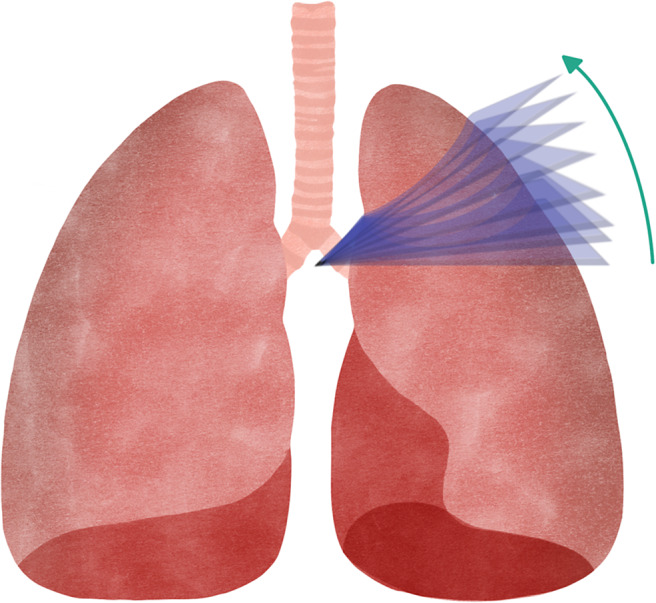
5-Axis dynamic slicing where you can see the constant change of planes to generate the structure in the most optimal way.

**Figure 10.**
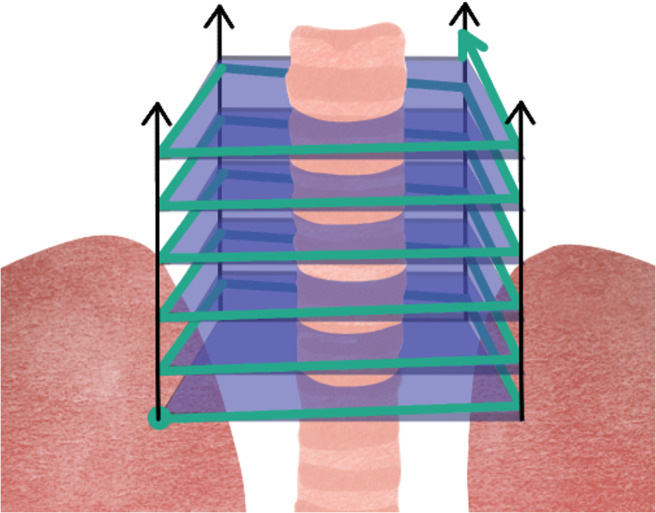
Helical slicing where it can be seen that the trajectory is continuous and ascending.

**Figure 11.**
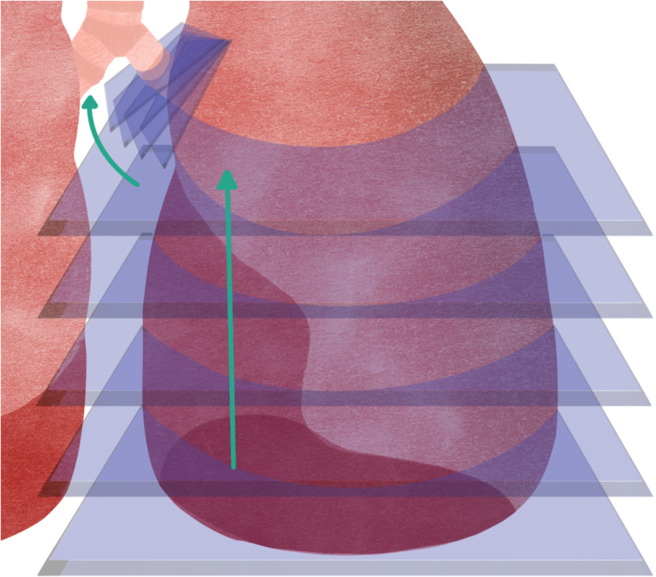
Mixed-layer adaptive slicing where the combination of uniform slicing and 5-axis dynamic slicing can be observed.

Compared to planar slicing algorithms, non-planar slicing algorithms allow more DOF at the cost of being much more complicated in terms of procedure and computational processing (Ref. 67) because, as the printing is not limited to a single plane, it is necessary to determine the toolpath of the print head very well to avoid collisions in its path (Ref. 62). All these techniques require the 3D bioprinter hardware to be able to print in more than one plane and to have at least 5 DOF with computerised robotic assistance, in addition to higher computing power (Ref. 68). The most common types of non-planar slicing are:

- **Curved-Layer Slicing (Figure 7):** the 3D model is interpreted in curved layers instead of flat layers, achieving a higher contour fidelity of the final object and reducing the need to include structural supports (Ref. 69).
- **Conformal Slicing (Figure 8):** the cuts are conformal to the preconstructed layer/substrate, so they can be flat or non-flat (Ref. 70). It allows printing a 3D structure on a free-form surface on which slices and layering are performed, as the z-axis coordinate changes continuously on the same layer, depending on the complexity and topology of a slice surface (Ref. 71). It requires the use of more sophisticated algorithms and GPU demanding software to translate 2D patterns to non-planar 3D surfaces (Ref. 71).
- **5-Axis Dynamic Slicing (Figure 9):** uses a multitude of planes to do the slicing for the different sections of the 3D model, selecting the cutting plane that best suits the needs, presenting a method that significantly reduces the support structures and is not very costly in terms of time and processing capacity that optimises the final result (Ref. 72).
- **Helical Slicing (Figure 10):** forms a single continuous 3D toolpath and eliminates seam defects, where the geometry is initially cut into flat slices and then, using two consecutive flat slices, direction vectors are constructed from the current layer to the next layer (Ref. 73).
- **Mixed-Layer Adaptive Slicing (Figure 11):** combines planar and non-planar slicing methods to optimise the printing process for different target subvolumes. This method takes advantage of the benefits of planar splicing and non-planar slicing to present a much higher quality result following a much more suitable manufacturing process (Ref. 69).

Some of these algorithms require a bioprinter of at least 5 DOF, such as the RoboPrint developed by Acoustic Robotic System Lab and Maxon, or 6 DOF, such as BioAssemblyBot by Advanced Solutions.

The choice of each type of slicing depends on both the technology available and the requirements of the result.

### Relationship between slicing approaches and cell viability

Slicing plays a fundamental role in the toolpath that the bioprinter will follow during the bioprinting process. From the process of converting a 3D model to STL format, a loss of topological fidelity can occur (Ref. 74), decreasing the accuracy of the bioprinting. To solve this, several algorithms can be used to generate different toolpath patterns depending on the part of the 3D model, either on the outside (contour) or the inside (infill density) (Ref. 75), allowing one to choose the most appropriate one for each case and improving the final result.

Infill density is a critical parameter in terms of structure and porosity. A construct with high infill density will exhibit higher mechanical properties and fidelity than structures with lower infill density, which is key to matching these properties to the desired fabric type (Ref. 76). In 3D bioprinting, the infill density determines the degree of porosity of the interior, which directly affects how the cells will interact with the element, and can be defined as (1 – fill density ratio) (Ref. 76).

Designing 3D models considering the porosity of the materials determines the degree of permeability and transport of nutrients, metabolic products, oxygen and, especially, the degree of cell migration along the final construct (Refs 77, 78).

Cells interact with their environment to a high degree of detail. These interactions shape their phenotype depending on very specific factors such as the presence of pores, pore size and the curvature of their substrate, among others (Ref. 79). Each cell type and tissue has its own porosity and pore size requirements. Fibroblasts, for example, exhibit optimal proliferation values in tissues with pores of 200–250 μm and 86% porosity (Ref. 80). Osteoblasts, on the other hand, show good growth in small pore sizes (40 μm), while larger pore sizes (100 μm) provide greater migration capacity (Ref. 81). Chondrocytes show a greater capacity for proliferation in scaffolds with pore sizes between 250 μm and 500 μm (Ref. 82). Although each cell type possesses a preference for a range of pore sizes for optimal proliferation, it has been suggested that a suit-all pore size would be in the range of 100–700 μm (Ref. 78).

In the case of curvature, it has been described that a concave geometry could favour the formation of aggregates with cell-to-cell connections, accelerating the formation of blood vessels (Ref. 83).

It should be noted that the bioprinting process, from the acquisition of medical images to the creation of bioprinted tissues, introduces several cumulative errors that can cause the final product to differ from the initial 3D model. First, the spatial resolution of the original DICOM images, usually from CT or MRI scans, directly affects the quality of the resulting 3D model (Ref. 56). In addition, segmentation and smoothing during 3D model generation can remove important details, reducing the fidelity of the model to the original anatomy (Ref. 84). The slicing process, where the model is converted into layers for printing, also introduces variations due to parameters such as layer resolution and model orientation, which can alter the final geometry (Ref. 85).

The quality of the mesh also influences the simulation and functionality of the final model. Research in airway airflow simulation indicates that a mesh with poorly distributed elements can lead to significant errors in predictions, underlining the importance of optimising each stage of medical image processing for bioprinting (Ref. 59).

Finally, during bioprinting, factors such as viscosity, osmotic pressure, injectivity, rheological properties, bioink surface tension, print flow control, process-induced mechanical forces and in situ cross-linking mechanisms can affect the fidelity of the bioprinted tissue (Ref. 86) introducing additional errors that affect both cell shape and viability.

The accumulation of these sources of error throughout each step produces a significant deviation between the initial digital model and the final bioprinted tissue, so carefully optimising each step of the process is key to reducing these variations.

## Parameter optimisation in biological models bioprinting

Once the image has been processed by *slicing* in the most appropriate way and considered what bioprinter is available, it is necessary to adjust the technical parameters that influence both the structural precision and the viability and functionality of the bioprinted cells and tissues. Thus, bioprinting parameters are defined as those settings or firmware inputs necessary to produce bioprinted structures in an appropriate way (Ref. 87).

The range of values suitable for bioprinting depends mostly on the type of bioink used and their physicochemical properties. Therefore, it is important to extensively characterise it before determining which parameters to adjust in the bioprinter before starting the bioprinting process. In the case of extrusion bioprinters, the most widely used due to their versatility, relatively low cost and capacity to generate thicker and bulkier structures, the main parameters to be adjusted are:

1. **Extrusion pressure:** is defined as the force applied to the bioink to push it through the nozzle of a bioprinter during the printing process. During the extrusion process, cells are exposed to mechanical forces of different types, which can play an important role in their differentiation, motility and growth control (Ref. 88). Of the different forces to which the cells are exposed, the one that has the greatest impact on them is shear stress, and is the main cause of cell death (Ref. 8). Therefore, because a higher pressure triggers a higher shear stress in extrusion bioprinting, causing more cell death (Ref. 89) it is advisable to reduce the pressure to the minimum allowed by the rheological properties of the bioink (Ref. 90).
2. **Printing speed:** is the speed at which the nozzle moves over the bioprinter bed and is defined as the flow rate divided by the feed rate. It is a dominant factor in printing results, affecting the height, width and thickness of the printed structures (Ref. 91). It is directly related to the previous parameter, as higher extrusion pressure requires higher printing speed, so speed adjustments must be made to minimise shear stress and maintain cell integrity (Refs 92, 93).
3. **Nozzle diameter:** nozzle diameter affects bioprinting by affecting the shear stresses caused by the internal pressure of the extruder nozzles, which affects printability and cell viability (Ref. 94). Thus, reducing the nozzle diameter in bioprinters increases the shear stress, which can lead to cell death due to mechanical damage (Ref. 95).
4. **Nozzle geometry:** nozzle geometry, including radius, length and angle of convergence, significantly influences shear stress, which, in turn, influences cell viability (Refs 96, 97). For example, cylindrical nozzles tend to generate the lowest shear stress, but this stress is maintained over a longer period, which can reduce cell viability (Ref. 97) while conical and truncated conical nozzles can increase the flow rate and reduce the dispensing pressure (Ref. 96). On the other hand, coaxial nozzles allow the creation of microchannels within the printed structures, improving cell viability by facilitating nutrient delivery (Ref. 98).
5. **Temperature:** influences bioink viscosity, cell viability and structural accuracy. Proper temperature control ensures that the bioink maintains a suitable viscosity for extrusion, guarantees cell survival (usually around 37 °C) and regulates the gelation of the biomaterials used, which is essential to maintain the printed structure without deformation. It also affects the final resolution and quality of the bioprinted tissues (Ref. 99).
6. **Layer height**: critical in determining the resolution and accuracy of printed structures, as well as the cell viability and mechanical integrity of the tissue. Thinner layers allow for greater precision and definition in detail. (Ref. 19), but may increase printing time and generate higher mechanical stresses on cells, while thicker layers speed up the process and reduce cellular stress, but may compromise resolution and homogeneity of tissues (Ref. 100). The balance between layer height, resolution and cell viability is key to the success of the process.

## Materials and cells used in bioprinting

The appropriate selection of materials and cells depends on the tissue to be bioprinted. Choosing the right components is essential for the successful reproduction of complex biological structures. Biomaterials used as bioinks must meet specific requirements, such as being biocompatible, promoting cell adhesion and possessing mechanical and functional properties comparable to the target tissue. These materials can be derived from natural sources, synthetic sources or combinations of both (Ref. 101). In addition, selected cells must be able to proliferate, differentiate and organise themselves into 3D architectures that mimic the functions of the tissue or organ to be bioprinted. To this end, the interaction between bioinks and cells is key, ensuring that the printed structure maintains its viability and functionality, both during the printing process and after implantation or maturation (Ref. 102). The selected bioink should be tested for its compatibility with the growth and function of a particular cell type and, therefore, for the development of a particular tissue. Table 2 exemplifies successful combination of recreated tissues with cell types.

**Table 2.** Bioprinting of tissues and the types of bioinks and cells used

Tissue type	Bioink	Cell type	Bioprinting method	References
Cartilage	dECM/Alginate	Adipose-derived mesenchymal stem cells (ASC)	Extrusion (BioX)	(Ref. 61)
Bone	GelMA/Bentonite	Bone Marrow Stromal Cells (BMSCs)	Extrusion (Bio-architect X)	(Ref. 62)
Cardiac tissue	GelMA/HAMA	Cardiac fibroblasts and cardiac myofibroblasts	Extrusion (BioX6)	(Ref. 63)
Vascular tissue	GelMA/Hyaluronic acid	Primary human foreskin fibroblasts (HFFs), Primary human aortic smooth muscle cells (hASMC), Human umbilical vein endothelial cells (HUVECs), murine fibroblasts L929	Inkjet (3D Bioplotter)	(Ref. 64)
Liver tissue	GelMA	Primary mouse hepatocytes (PMHs) and endothelial cell line C166	Extrusion (BioMaker2)	(Ref. 65)
Skin	GelMA/ carbon nanofiber nanoparticles (CNF NPs)	Normal human primary human fibroblasts (nHDF)	Extrusion (BioX)	(Ref. 66)
Neural tissue	LAMININK 521 (CELLINK)	U–251 and DK-MG Human glioblastoma multiforme cell line	Extrusion (BioX)	(Ref. 67)
Lung tissue	Sodium alginate/chitosan/gelatin/agar-methylcellulose	Epithelial lung cancer cell line H69AR	Extrusion (BioX)	(Ref. 68)
Renal tissue	Fibrin hydrogel	Primary murine tubular epithelial cells (pmTEC) and Human umbilical vein endothelial cells (HUVEC)	Extrusion (BioScaffolder 3.1)	(Ref. 69)
Skeletal muscle	RGD alginate	C2C12 mouse myoblasts and primary bovine muscle stem cells (MuSCs)	Extrusion (Allevi 2)	(Ref. 70)
Pancreatic tissue	LAMININK 521 (CELLINK)	Cap neural crest stem cells (BCs) and pancreatic islets (PIs)	Extrusion (BioX)	(Ref. 71)
Gut tissue	GelMA-PEGDA	Caco–2 C2BBe1, mucus-producing HT29-MTX cells and Human intestinal fibroblasts (HIF) primary cells	Light-based bioprinting (SOLUS 3D)	(Ref. 72)
Corneal tissue	dECM/collagen	Adipose tissue-derived stem cells (hASCs)	Inkjet (3D Bioplotter)	(Ref. 73)
Periodontal tissue	GelMA	Primary human periodontal ligament cells (hPDLCs) and human gingival fibroblasts (hGFs)	BioX	(Ref. 74)
Endometrial tissue	Alginate/collagen	Adipose-derived mesenchymal stem cells (ADSCs)	Extrusion (Baobab Root)	(Ref. 75)

## Functional test of bioprinted constructs

Testing cell viability and functionality of bioprinted constructs is an essential step to ensure the generated structures perform as expected. During the bioprinting process, cells are exposed to changing environmental conditions (e.g. temperature, viscosity, mechanical forces) that can impact their viability and function (Refs 103, 104). There exist different methods to assess cell viability. However, they were originally designed for 2D conditions and they do not often perform well in 3D conditions. We propose the combination of methods for an adequate assessment of cell viability in these conditions, including (Refs 105, 106, 107):

- **Live/Dead staining:** a methodology for the assessment of cell viability in both 2D and 3D cultures. It is widely employed through the combination of two fluorogenic compounds: calcein acetoxymethyl ester (calcein AM) and propidium iodide (PI). Differentiation between viable and non-viable cells is based on cell metabolic activity and plasma membrane integrity. The major component of this assay is Calcein AM, a non-fluorescent molecule hydrolysed in viable cells by intracellular esterases into calcein, a highly fluorescent substance. This process occurs exclusively in cells with active metabolism and intact membranes, as only living cells possess the requisite enzymatic activity to release the acetoxymethyl group from Calcein AM and convert it into a fluorescent compound (Ref. 108). The emission wavelength of calcein is approximately 515 nm, which permits its detection through the use of fluorescence microscopy or flow cytometry (Ref. 109).
  In contrast, PI is a marker that only permeate to cells with compromised plasma membranes, which is indicative of non-viable cells. PI is positively charged, rendering it unable to cross intact cell membranes. However, in damaged cells, it is capable of passing through and binding to cellular DNA and RNA (Ref. 110) via electrostatic interactions with the phosphate groups of nucleic acids. The intercalating bond between propidium iodide and DNA markedly enhances the fluorescence of the latter, resulting in the emission of light at a wavelength of approximately 617 nm upon excitation.
  The live/dead staining technique is widely used to assess viability in conventional cell cultures. Its use in 3D cell cultures can be compromised due to limited penetration of the compounds, therefore to obtain a more accurate quantitative assessment of viability in 3D models, it is recommended to combine this technique with other complementary methods to improve the accuracy of in-depth detection (Ref. 107).
- **Alamar Blue (AB) assay:** a widely used technique for assessing the metabolic activity of cells based on the reduction of the non-fluorescent compound resazurin to the fluorescent compound resorufin by enzymatic reductases using reducing metabolites such as NADH, NADPH or FADH (Refs 111, 112). The resorufin produced can be quantified by absorbance, measured at a wavelength of 570 nm, or by fluorescence, using an excitation wavelength of 540 nm and an emission of 590 nm (Ref. 111), allowing sensitive and quantitative detection of cell activity.
  In bioprinting applications, cell encapsulation within bioprinting gels can impact the assay’s performance. Key variables that may affect assay effectiveness include the gel’s concentration, thickness and water content. For instance, in hydrogels with high water content, such as those based on collagen, AB dye dilution can occur, reducing assay performance by up to 25% (Ref. 113). To mitigate these effects, it is recommended to calibrate assay conditions according to the specific properties of the bioprinted material being used.
- **PicoGreen Assay**: this technique measures the DNA content of cells encapsulated in the gel, which can be used to allow an estimation of their proliferation. The technique involves breaking the cell membranes to release the DNA and measuring its concentration by fluorescence. Therefore, is not suitable for continuous monitoring of cell proliferation.

Therefore, for optimal gel/3D structure characterisation, the effects of hydrogel structure, water content and porosity have to be considered when selecting a cell viability assay.

## Challenges and limitations for the clinical translation of 3D bioprinting

The clinical application of bioprinting still faces numerous challenges that must be addressed. With regard to immunological safety and scaffold immunogenicity, it is known that hydrogels and scaffolds can trigger innate immune responses and capsular fibrosis, impairing graft integration, perfusion and function (Ref. 114). In certain natural bioinks, impurities are critical determinants. For example, alginate is often contaminated with endotoxins, proteins and polyphenols capable of inducing inflammation and cellular overgrowth within capsules, making purification essential to mitigate these effects (Ref. 115). Similarly, decellularised ECM-derived matrices provide biomimetic signals, but residual immunogenicity largely depends on meeting minimal decellularisation criteria, including the content and size of nuclear and mitochondrial DNA fragments, the presence of reactive oxygen species (ROS), and fragmented ECM components such as hyaluronic acid or fibronectin (Ref. 116).

Sterilisation is another critical factor in the development of new bioinks for clinical use, as it can significantly alter key properties. For instance, GelMA sterilisation by autoclaving or ethylene oxide reduces stiffness, whereas gamma irradiation increases stiffness, alters pore size, and may compromise sol–gel transition, printability and 3D cell viability (Ref. 117). Consequently, sterilisation procedures for each bioink must be systematically evaluated alongside other characterisation tests to fully understand the modifications introduced during processing.

The viability of bioprinted tissues also remains constrained by limited oxygen and nutrient diffusion in the absence of a fully functional vascular network. Although printing strategies that rely on diffusion can produce perfusable channels and enhance molecular transport within constructs, maintaining stable perfusion and effective vascular integration in vivo remains a major hurdle for the development of volumetric tissues (Ref. 118).

The choice of cell source also represents a key translational barrier. Autologous cells minimise the risk of immune rejection but complicate logistics and extend application timelines. Allogeneic cells offer greater scalability but may elicit donor-specific antibodies and consequent immune responses (Refs 119, 120). Induced pluripotent stem cells (iPSCs) present another alternative, although potential links to tumorigenicity have been suggested, underscoring the need for further studies and rigorous genomic and functional quality controls before clinical implementation (Ref. 121).

Finally, one of the most critical barriers to clinical translation is the lack of standardisation, quality control and reproducibility of bioinks. Batch-to-batch variability in natural polymers and ECM-derived materials, the degree of functionalisation, the type of crosslinking agent, the light source used in photopolymerisation and rheological properties, among others, directly affect viability, printing fidelity and mechanical performance. Therefore, the establishment of robust bioink standardisation protocols and reproducible quality control systems is essential to achieve successful clinical translation of bioprinted constructs.

## Conclusion

The workflow for generating 3D models from DICOM medical images and their bioprinting represents a significant advance in addressing critical challenges in medicine, such as organ shortages and the need for more accurate models for biomedical research. However, each step of the process – from image acquisition to slicing and bioprinting – carries the potential for cumulative errors that can compromise both the accuracy and functionality of the resulting models.

Factors such as the resolution and quality of the initial images, together with the conversion to STL format, are decisive in the accuracy of the 3D model. Slicing and printing parameters also play a crucial role in the final structure and cell viability of bioprinted tissues.

Optimising each of these stages is essential to minimise deviations between the digital model and the bioprinted tissue, thus improving the quality of the generated scaffolds and their applicability in clinical and research contexts. As technological advances in bioprinting and image processing methods continue to develop, the accuracy of these models is expected to increase, enabling the creation of more complex and functional tissues, with great potential to contribute to regenerative and personalised medicine.

Furthermore, 3D models generated from medical images not only have applications in bioprinting but have also proven to be valuable in the simulation of physiological functions. For example, they have been successfully used in the reproduction of forced spirometry tests, simulating airflow dynamics in the airways of individual patients. These simulations constitute a powerful tool for the evaluation and personalisation of medical treatments, expanding the scope of 3D models beyond the realm of bioprinting.
